# Regulation of Signal Transduction by Glutathione Transferases

**DOI:** 10.1155/2012/137676

**Published:** 2012-10-09

**Authors:** Julie Pajaud, Sandeep Kumar, Claudine Rauch, Fabrice Morel, Caroline Aninat

**Affiliations:** Inserm, UMR991, Foie, Métabolismes et Cancer, CHU Pontchaillou, 35033 Rennes, France

## Abstract

Glutathione transferases (GST) are essentially known as enzymes that catalyse the conjugation of glutathione to various electrophilic compounds such as chemical carcinogens, environmental pollutants, and antitumor agents. However, this protein family is also involved in the metabolism of endogenous compounds which play critical roles in the regulation of signaling pathways. For example, the lipid peroxidation product 4-hydroxynonenal (4-HNE) and the prostaglandin 15-deoxy-Δ12,14-prostaglandin J_2_ (15d-PGJ_2_) are metabolized by GSTs and these compounds are known to influence the activity of transcription factors and protein kinases involved in stress response, proliferation, differentiation, or apoptosis. Furthermore, several studies have demonstrated that GSTs are able to interact with different protein partners such as mitogen activated protein kinases (i.e., c-jun N-terminal kinase (JNK) and apoptosis signal-regulating kinase 1 (ASK1)) which are also involved in cell signaling. New functions of GSTs, including S-glutathionylation of proteins by GSTs and ability to be a nitric oxide (NO) carrier have also been described. Taken together, these observations strongly suggest that GST might play a crucial role during normal or cancer cells proliferation or apoptosis.

## 1. Introduction

Glutathione transferases (GSTs) represent a major cellular defence system; they constitute a multigene family divided in seven families (Alpha, Mu, Pi, Theta, Sigma, Zeta, and Omega) with functions ranging from detoxification to biosynthesis and cell signaling [1, 2]. The most extensively investigated role of GSTs is their function of detoxification enzymes, where they catalyse the nucleophilic attack of glutathione (GSH) on electrophilic substrates. This mechanism allowed to protect a variety of cell components (protein, lipid, DNA) against reactive molecules such as electrophilic metabolites formed after xenobiotics phase I metabolism or endogenous *α*,*β*-unsaturated aldehydes and hydroperoxides formed as secondary metabolites during oxidative stress. 

GSTs are also involved in metabolism of endogenous lipid mediators which influence diverse-signaling pathways. Among them, the 15-deoxy-Δ^12,14^-prostaglandin J_2_ (15d-PGJ_2_) regulates the activity of three transcription factors playing a central role in stress response, differentiation and proliferation: the peroxisome proliferator-activated receptor **γ**(PPAR**γ**), the nuclear factor-erythroid 2 p45-related factor 2 (Nrf2), and the nuclear factor **κ**B (NF-**κ**B) [2]. Another one, the endogenous lipid peroxidation product 4-hydroxynonenal (4-HNE) is also believed to act as an intracellular signaling molecule [3]. Therefore, its conjugation with glutathione by GSTs will influence a number of pathways. Indeed, like 15d-PGJ_2_, 4-HNE can stimulate gene expression through Nrf2 and prevent activation of NF-**κ**B by inhibiting I**κ**B phosphorylation. It has also been reported to modulate several cell-surface receptors, to activate epithelial growth factor receptor and platelet-derived growth factor-**β**receptor, and to upregulate transforming growth factor receptor **β**1 [4]. Altogether, these observations suggest that GSTs, which are involved in 4-HNE and 15d-PGJ_2_ metabolism, will certainly influence many signal transduction pathways and modulate cell survival and proliferation.

During the last decade, research on GSTs has unravelled yet another major function, namely a role in regulating cellular signaling by forming protein-protein interactions with critical proteins involved in controlling stress response, apoptosis, and proliferation. For example, the ligand-binding capacity of GST results in the negative regulation of signaling pathways through sequestration of protein kinases. Adler et al. [5] published the first study showing that mouse GSTpi interacts with the protein kinase c-jun N-terminal kinase (JNK). Dissociation of this complex by different types of stress leads to the activation of JNK and phosphorylation of its substrate, the transcription factor c-jun. Thereafter, other interactions have been identified and their implication in regulation of different biological processes has been demonstrated.

Another interesting function of GST, and especially of GSTPi, involved the regulation of a posttranslational modification of proteins, the S-glutathionylation and its implication in the protection against oxidative damage and the control of the redox signaling pathway. S-glutathionylation is characterized by the conjugation of GSH to low-cysteine sulfydryl or sulfonic-acid moieties in target proteins. Several studies have shown that various intermediates of signaling pathways controlling the survival/apoptosis mechanisms (p53, caspase 3,…) could be S-glutathionylated [6, 7]. Interestingly, these modifications seemed to modulate their activities.

Last, but not least, a thrilling new concept of NO stockage by GSTs have been brought up [8]. Indeed, several GSTs, and especially GSTP1-1, could bind NO under dinitrosyl iron complexes (DNICs). This binding seems to protect cells against high levels of DNICs, which are known to inhibit glutathione reductase, and to limit the peroxinitrite formation [9]. 

Taken together, these observations strongly suggest that GST might play a crucial role during normal, or cancer-cells proliferation or apoptosis. In this paper, we will focus on the major findings regarding the different modes of action of GST to regulate cell signaling, and we will give some examples demonstrating the involvement of GSTs in the regulation of hepatocyte proliferation and apoptosis.

## 2. 4-HNE, Cell Signaling, and GSTA4 

4-HNE is a major product of the lipid peroxidation process that is characterized by peroxidative decomposition of polyunsaturated lipids. The mitogen-activated protein kinase (MAPK) pathways involved in cellular stress responses appear to be particularly sensitive to 4-HNE [4]. Indeed, the ability of 4-HNE to initiate increases in tyrosine phosphorylation is involved in the activation of c-jun N-terminal kinases (JNK) and p38 [10]. Both of them can regulate several transcription factors involved in cellular responses including cell proliferation, inflammatory responses, proteasome-mediated protein degradation and apoptosis. Many studies underlined concentration-dependent effect of 4-HNE on cell signaling pathways. A moderately high concentration of 4-HNE can induce apoptosis, differentiation, and affect activation of adenylate cyclase, JNK, protein kinase C, and caspase 3 [11, 12]. In contrast, a low concentration of 4-HNE can induce cell proliferation. Another study confirmed that 4-HNE has a dose-dependent effect, and a distinction could be made between a supraphysiological concentration (100 *μ*M), which was primarily cytotoxic and a physiologicalrange (below 10 *μ*M) modulating cell growth [13]. These effects consist in a transient inhibition of the initial phase of cellgrowth, which under optimal conditions (in presence of serum) was followed by a period of increased proliferation, compared to untreated control cultures, until confluence was attained [13]. 

4-HNE also inhibits the expression of cyclin D1, D2 and A and, consequently, the activity of cyclin-dependent kinase 4/6 (Cdk4/6) and Cdk2 [14]. Interestingly, these Cdk-cyclin complexes are involved in the phosphorylation of retinoblastoma proteins, and therefore their partial inactivation, allowing the transcription of E2F-controlled genes and the progression in S phase. Moreover, 4-HNE upregulates the expression of p21^waf1^ which is involved in the negative regulation of cyclin-Cdk complex protein kinase activities [15]. These findings show that 4-HNE can orchestrate the simultaneous expression of many different genes involved in the control of cell proliferation [16].

These observations clearly demonstrate that 4-HNE intracellular amount must be tightly controlled to prevent cellular damages and/or to regulate stress-response signaling. Although different enzymes such as alcohol dehydrogenase, aldolase reductase, or aldehyde dehydrogenase are involved in the metabolism of 4-HNE, the majority of 4-HNE is metabolized by GST, via its conjugation to GSH, which promotes its detoxification [18, 19]. In the liver, Kupffer and stellate cells have the capacity to metabolize 4-HNE, but to varying degrees compared to hepatocytes (100 times less efficiently for Kupffer cells than hepatocytes). The main GST involved in 4-HNE detoxification is GSTA4 [20, 21]. Interestingly, mGSTA4 was induced *in vivo* and in cultured hepatocytes by tumor necrosis factor *α* (TNF*α*), interleukin-6 (IL-6), and epidermal growth factor (EGF) [22]. All these factors that play crucial roles in hepatocyte survival and proliferation during liver regeneration. Moreover, *mGsta4* gene expression was increased at 1 and 24 hour post-partial hepatectomy (PH) compared with normal and sham-operated animals while a 3-fold increase in 4-HNE levels was observed 1 hour after PH [22].

Altogether, these studies demonstrate that the intracellular concentration of 4-HNE appears to be crucial for cell cycle signaling and may be a determinant for the signaling during differentiation, proliferation, transformation, or apoptosis. Importantly, the intracellular concentrations of 4-HNE are regulated by the action of GSTA4-4, which conjugates 4-HNE to GSH.

## 3. Modulation of 15d-PGJ_**2**_ Signaling Pathway by GST

Prostaglandins (PG) are lipid compounds enzymatically derived from arachidonic acid that is released from the cell membrane phospholipids by phospholipase A2. Arachidonic acid is first metabolized by cyclooxygenase in PGG_2_, which in turn is transformed in PGH_2_ by PGH_2_ synthase. PGH_2_ is then conversed in other prostagladins (PGE_2_, PGF_2_ *α*, PGI_2_, thromboxanes, PGD_2_) by several specific synthases. These mediators are autocrine or paracrine molecules with local activities and involved a large panel of functions including inflammation, neuronal plasticity, and platelet aggregation. Some of them (PGE_2_, PGD_2_, and PGF_2_ *α*) are secreted and act by binding to a plasma membrane receptor. 

**Figure 1:**
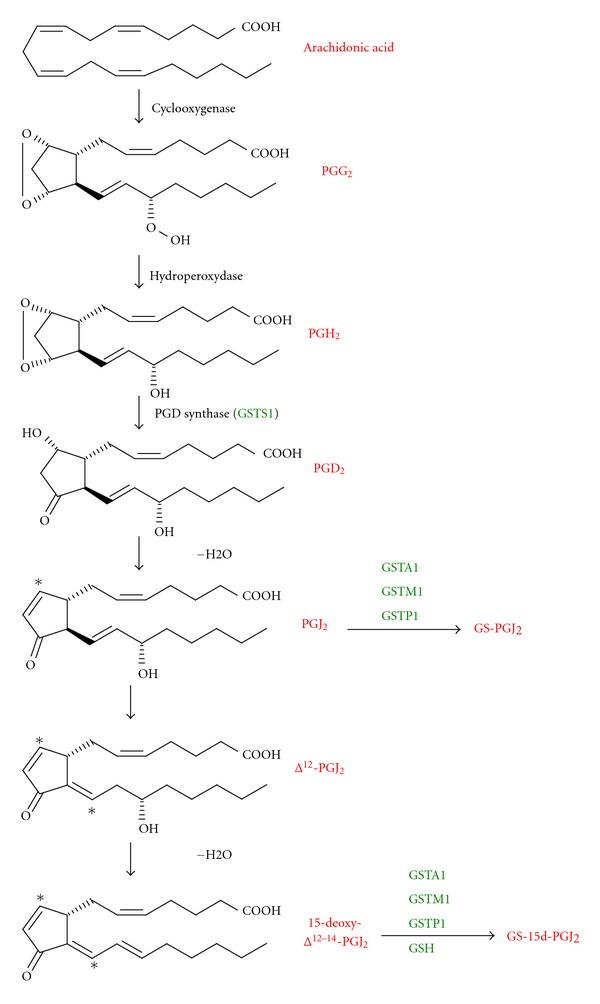
The prostaglandin biosynthetic pathway (adapted from [1]) 15-deoxyΔ^12-14^-PGJ_2_ is a metabolite derived from arachidonic acid. Several GSTs are implicated in the regulation of its formation: GSTS1 metabolized PGH2 in PGD2; GSTA1, GSTM1, and GSTP1 conjugated GSH to PGJ_2_ and 15-deoxyΔ^12-14^-PGJ_2_. This conjugation led to the regulation of various transcription factors (PPAR*γ*, NF-*κ*B, and Nrf2).

Among the prostaglandin species, 15-deoxy-Δ^12-14^ prostaglandin J_2_ (15d-PGJ_2_) is a downstream metabolite of PGD_2_ that acts by binding to intracellular receptors or transcription factors (Figure 1) [23]. Indeed, this compound owns an electrophilic *α*,*β*-unsaturated carbonyl group in its cyclopentenone ring, which can interact with cellular nucleophile groups such as thiols present in glutathione or cysteine. 15d-PGJ_2_ biological effects are multiple. For example, it is a natural activating ligand of PPAR*γ* [24]. After activation, PPAR*γ* is heterodimerized with Retinoid X Receptor (RXR) leading to the induction of PPRE-driven gene expression. In the liver, the level of PPAR*γ* is low, however it is implicated in several pathologies and its activation leads to a diminution of hepatocellular cancer growth by induction of cell apoptosis [25, 26]. Interestingly, binding of 15d-PGJ_2_ to PPAR*γ* in mouse liver results in the induction of hepatocyte growh factor (HGF) [27] and HGF induction is known to increase apoptosis and to decrease DNA synthesis in HepG2 [28]. A recent study has also linked the antineoplastic role of 15d-PGJ_2_ in the HBV-associated HCC (Hepatitis B Virus-associated Hepatocellular Carcinoma) growth and the activation of PPAR*γ* [29]. 

Furthermore, two different studies have suggested a potential role of 15d-PGJ_2_ in hepatic cell proliferation. Cheng et al. [30] reported that the 15d-PGJ_2_ was involved in the growth, cell cycle, and differentiation of hepatic oval cells, raising the possibility that the PPAR*γ* ligands may regulateliver regenerationand hepatocarcinogenesis. In a second study, Yamamoto et al. [31] demonstrated that, during rat liver regeneration, the number of PPAR*γ*-stained hepatocytes decreased 24 h after partial hepatectomy and increased in the late phase ofliver regenerationcompared to the sham-operated group. Moreover, the peaks of serum 15d-PGJ_2_ level and hepatic PPAR*γ* expression coincided with the late phase ofliver regeneration [31].These authors concluded that the PPAR*γ*/15d-PGJ_2_ system may be one of the key negative regulators of hepatocyte proliferation and may be responsible for the inhibition oflivergrowth in the late phase ofliver regeneration.

15d-PGJ_2_ has also been shown to inhibit the NF-*κ*B signaling pathway [32]. In cells, NF*κ*B is associated with I*κ*B proteins in the cytoplasm in an inactive complex. After proinflammatory or growth factor stimuli, phosphorylation of I*κ*B by I*κ*B kinase (IKK) leads to its proteasomal degradation. These conditions allow the release of NF-*κ*B, its phosphorylation and its translocation in the nucleus where, alone or in combination with other transcription factors, it induces target gene expression [33, 34]. The role of NF-*κ*B in controlling cell cycle regulators, and more particularly cyclin D1, has been observed in investigations that used the I*κ*B “super repressor” in order to inhibit NF-*κ*B activity [35]. These findings suggest an important role for NF-*κ*B in the regulation of cell cycle. Furthermore, NF-*κ*B with upstream participation of TNF*α*, signaling through TNF receptor 1 (TNFR1) together with IL-6 and signal transducers and activators of transcription 3 (STAT3) is required for initiation ofliver regeneration [36]. Several studies have shown that 15d-PGJ_2_ is able to inhibit the NF-*κ*B, targeting IKK by a covalent binding on a cysteine 179 (Cys-179) [32, 37]. 15d-PGJ_2_ also directly inhibits binding of NF-*κ*B to DNA-specific sequences by modifying the NF-*κ*B Cys-38 [32]. Furthermore, Okano et al. [38] have observed that 15d-PGJ_2_ suppressed NF-*κ*B activation through independent PPAR*γ* mechanisms in a hepatic cell line (SK-Hep1 cells). Interestingly, the same effects were observed in HepG2 cells, however, in this cell line the mechanism seems to involve the PPAR*γ* activation. 

15d-PGJ_2_ can also stimulate Nrf2-mediated induction of gene expression through the antioxidant response element [39, 40]. Indeed, 15d-PGJ_2_ is able to modify cysteine residues in the cytoskeleton-associated protein Keap1 (Kelchlike ECH-associated protein 1), and thus overcomes the ability of Keap1 to target Nrf2 for proteasomal degradation [41]. Therefore, conjugation of 15d-PGJ_2_ with GSH abolishes its ability to modify Keap1. The regulation of Nrf2 by 15d-PGJ_2_ might have important consequences in liver regeneration. Indeed, Beyer et al. [42] demonstrated impairedliver regenerationinNrf2knockout mice and revealed novel roles ofNrf2in the regulation of growth factor signaling and in tissue repair. The same group showed that Nrf2 controls insulin receptor signaling in the regenerating liver [43]. Finally, a recent work has demonstrated thatNrf2recognized a functional ARE (antioxidant responsive element) in the promoter of Notch1 that regulates processes such as proliferation and cell-fate decisions [39]. In this study, the authors have reported a functional role for this cross talk between the two pathways and show a delayedliver regenerationafter partial hepatectomy in Nrf2 knockout mice that was rescued by reestablishment of Notch1 signaling. Taken together, these studies suggest that 15d-PGJ_2_ could also modulate liver regeneration through the regulation of Nrf2.

Different studies have shown that GSTs are able to regulate the level of 15d-PGJ_2_. Indeed, GSTs play a critical role at several levels in the synthesis and the degradation of this compound. GSTS1 has been identified as the prostaglandin synthase implicated in the production of PGD_2_ (Figure 1), the metabolicprecursorof 15d-PGJ_2_ [44]. On the other hand, GSTA1, GSTM1, and GSTP1 have been shown to catalyze the conjugation of PGJ_2_ with glutathione [45]. This conjugate is then eliminated by the MRP (Multidrug Resistance Protein) transporter. 15d-PGJ_2_ is also metabolized via conjugation with glutathione in HepG2 cells [46], however, this conjugation can be observed in presence or absence of GST suggesting that the level of GSH in cell could modulate the action of 15d-PGJ_2_ [47]. Kawamoto et al. [48] have observed that 15d-PGJ_2_ is able to induce the GSTP1 in the R34 rat liver epithelial cell line through binding of different proteins, including c-jun, to a responsive element present in the GSTP1 5′-flanking region. On the other hand, 15d-PGJ_2_ is able to directly posttraductionally modify GSTP1 to inhibit its activity. This covalent binding implicates alkylation of the Cys-47 and/or 101 [49]. Since GSTP1 is overexpressed in tumor cells and might be involved in anticancer drug resistance, 15d-PGJ_2_ binding to GSTP1 could lead to the development of irreversible inhibitors in anticancer therapy. Interestingly, the binding or sequestration of 15d-PGJ_2_ to GST is also observed with GSTM1a and GSTA1 and inhibits the transactivation of PPAR*γ* [50]. The ability of different GSTs to affect either synthesis, or elimination of 15d-PGJ_2_ places GSTs as central regulators in cell signaling mediated by this eicosanoid.

## 4. GST-Protein Interactions and Cell Signaling

**Figure 2:**
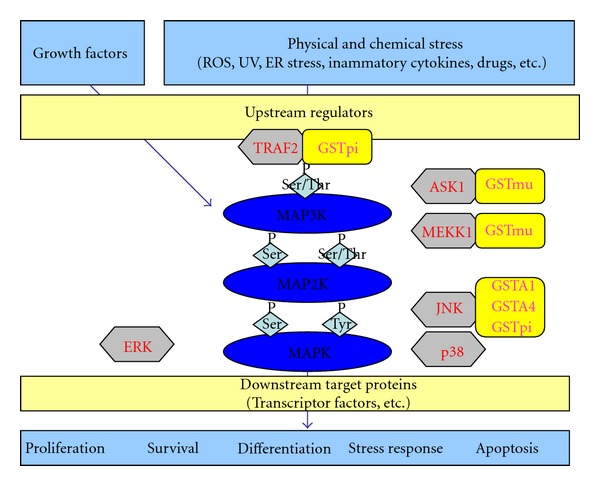
Scheme of the various interactions between GSTs and MAPK implicated in stress-signaling pathway (adapted from [17]). The mitogen-activated protein kinase (MAPK) family is composed of three types of kinases: MAP3K, MAP2K, and MAPK. In mammal, 3 major subgroups of MAPK are found: ERK, JNK, and p38. ERK is activated by proliferation and differentiation stimuli whereas JNK and p38 are preferentially activated by environmental stress. Upstream kinases (MAP3K, MAP2K) initiate activation of MAPK cascade in response to environmental changes and MAPK phosphorylate downstream targets such as transcription factors and generate appropriate biological response. Several GSTs are able to interact with various of these MAPK in nonstress conditions. Environmental stress leads to the disruption of these interactions and the activation of the signaling pathway. ROS: reactive oxygen species; UV: ultraviolet; ER: endoplasmic reticulum; TRAF2; TNF-receptor-associated factor 2; ASK1: apoptosis signal-regulating kinase 1; MEKK1:Mitogen-activated protein kinase kinase kinase1; JNK: c-Jun N-terminal kinase 1; ERK: extracellular regulated kinase.

Cells are continuously exposed to external or internal stress which trigger signaling pathways and lead to the activation of several biological processes such as cell proliferation, differentiation, apoptosis or stress response. Control of these different pathways involves upstream activation of three protein kinase families: MAP3K, MAP2K and MAPK. Regulation of these protein kinases is complex and the existence of stress sensors. In the last decade, literature brought up the idea that GSTs could play such a role (Figure 2). 

The first evidence for a direct interaction of a GST with another protein has been published by Adler et al. [5]. In this study, the authors demonstrated that mouse Gstpi interacts with JNK in mice 3T3/4A fibroblasts. Under a monomeric state, Gstpi acts as a direct JNK inhibitor in nonstressed cells by forming a complex with JNK and c-jun. Oxidative stress (UV, H_2_O_2_, etc.) induces the dimerization of GSTpi and activation of c-jun through its phosphorylation on Ser-63 and Ser-73 residues. Residues 194 to 201 (sequence SSPEHVNR) of Gstpi [4] and the C-terminal region of JNK [51] seem to be implicate in this interaction.

Subsequently, several other studies have corroborated this model. For example, Bernardini et al. [52] analyzed the correlation between the modulation of the GSTP1 expression, its dimerization and its catalytic activity following treatment of human leukemia Jurkat cells with agents known to induce apoptosis through a JNK-dependent signaling pathway. Results have shown that hydrogen peroxide (H_2_O_2_) and, to a lesser extent, etoposide lead to the activation of JNK pathway. This process was concomitant to the apparition of dimerized forms of GSTP1 owning disulphide bound between their Cys-47 and monomeric forms owning intrasubunit disulphide bound between Cys-47 and Cys-101. Furthermore, this dimerization is responsible for an inhibition of the GST activity which could be explained by the localization of these cysteines in the glutathione-binding domain of GSTP1. However, in a recent work, Gildenhuys et al. [53] have criticized this model. Indeed, using equilibrium folding and unfolding kinetic experiments as well as molecular modelling they brought the demonstration that binding with JNK involved the dimeric form of GSTP1-1. Thus, further works are necessary to determine the real mechanisms involved in these interactions. On the other hand, understanding of these processes is also complicated by the fact that different haplotypes of GSTP1 triggered different effects. Indeed, two common functional variants of GSTP1 have been identified at amino 105 (Ile-Val) and 114 (Ala-Val). These variants lead to the existence of four haplotypes: the wild type GSTP1∗A (Ile^105^ + Ala^114^), and three variants GSTP1∗B (Val^105^ + Ala^114^), GSTP1∗C (val^105^ + Val^114^) and GSTP1∗D (Ile^105^ + Val^114^). GSTP1∗A has been shown to be able to slowdown cell's proliferation whereas the GSTP1∗C haplotype had no impact on this endpoint [54]. Furthermore, GSTP1∗A seems to be able to protect cells from apoptosis through a JNK-independent pathway while for GSTP1∗C this effect seems to be JNK dependent [54]. More recently, Thévenin et al. [55], have observed a higher inhibitory effect of GSTP1∗C on the phosphorylated isoforms JNK 1 and 2 compared to GSTP1∗A suggesting that these interactions depend on the activation's state of JNK. They have also demonstrated that interaction of phosphorylated JNK is enhanced in presence of ATF2, another substrate of JNK involved in oncogenesis, and that ATF2 is needed for the interaction of inactived JNK with GSTP1.

*In vivo* studies have also been performed and shown that in GSTpi−/− mice, JNK activity is constitutively enhanced, at least in liver, lung, and fibroblasts, and that, in such conditions, JNK-signalling pathway is upregulated triggering an increase in AP-1 DNA binding and HO-1 mRNA expression [56]. More recently, Castro-Caldas et al. [57] have observed, in a mouse Parkinson's disease model induced by a neurotoxin, that GSTpi−/− mice are more sentitive than wild-type mice to this stress. Indeed, in the midbrain and in the striatum, GSTpi seems to play the role of an endogenous regluator of the JNK signalling pathway by directly interacting with JNK.

Noteworthy, the direct interaction of JNK with GSTs is not limited to the GSTPi family. Indeed, Romero et al. [58] have shown that GSTA1 interacts physically with JNK in caco-2 cells. They showed that GSTA1 levels were lower in preconfluent cells than in postconfluent cells and they observed that response of caco-2 cells to a sodium butyrate JNK-dependent apoptotic stimulus was more important in preconfluent cells. In a different study, Desmots et al. [59] have established a correlation between phosphorylation of JNK and mGSTA4 upregulation under oxidative stress conditions and demonstrated that mouse GSTA4 and JNK coimmunoprecipitate in liver tissue extracts suggesting that mGSTA4 might be also an endogenous regulator of JNK activity by direct binding. Furthermore, these authors showed that hepatic mGSTA4 is strongly increased during oxidative stress possibly via JNK pathway and during proliferation via MEK/extracellular signal-regulated kinase pathway. 

In 2001, Cho et al. [60] have shown by yeast two-hybrid technology that mouse GSTM1-1 is able to interact directly with ASK1, a protein kinase belonging to the MAP3K family. This interaction inhibits apoptosis signal regulated kinase 1 (ASK1)-mediated activation of JNK/SAPK signaling pathway induced by several stress stimuli such as H_2_O_2_ or UV when GSTM1-1 is overexpressed in cells. Therefore, it was suggested that GSTM1-1 has a role as an ASK1-repressor under unstimulated conditions. Furthermore, this role seems to be independent of the GST activity since mutant GSTM1-1 lacking catalytic activity also represses ASK1. The involvement of the C-terminal region of GSTM1-1 and N-terminal region of ASK1 in this interaction has been determined using truncated proteins [60]. Intriguingly, the same region of ASK1 interacts with thioredoxin (Trx) and it has been shown that, depending on the type of stress, ASK1 dissociates from GST or Trx suggesting the presence of a pool of ASK1-GSTM1-1 and ASK1-Trx complexes under unstressed conditions. Indeed, Dorion et al. [61] have observed that heat shock is able to disrupt the interaction between ASK1 and GSTM1 leading to the heat-shock-mediated p38 signaling activation, whereas no dissociations were observed between ASK1 and Trx under the same conditions. Furthermore, they observed that ROS exhibited the opposite effect, triggering dissociation between ASK1 and Trx with an activation of the p38 oxidative stress sensing pathway without any effect on the ASK1-GSTM1 complexes. Interestingly, Gilot et al. [62] have suggested that not only GSTM1, but also GSTA1 and GSTP1, could play a key role in regulation of ASK1 protein kinase activity in rat hepatocytes and thus on apoptosis.

GSTP1 is also able to block ASK1 activation by interacting physically with the Tumor necrosis factor receptor associated factor 2 (TRAF2) [63]. TRAF proteins associate with, and mediate the signal transduction from, members of the TNF receptor superfamily.For example, binding of TNF*α* on its receptors, TNF Receptor 1 or 2, leads to the homotypic aggregation of these receptors which results in the recruitment of several adaptors in the receptor cytoplasmic N-terminal domain. Among these adaptors, TNF-R1 associated death domain (TRADD) is able to recruit TRAF2 after TNF*α*-activation of TNF-Receptor 1, while a direct association between TNF-Receptor 2 and TRAF2 is observed. These interactions trigger activation of JNK and p38 signal pathways by a dissociation of the ASK1-Trx complex. Wu et al. [63] demonstrated that the binding of GSTP1 and TRAF2 triggers the suppression of TNF*α*-TRAF2-ASK1 signaling pathway activation. Similarly to the other interactions described previously, the activity of GSTP1 is not necessary for this binding, and the interaction between GSTP1 and TRAF2 is observed only in unstimulated cells.

Many other studies have confirmed the involvement of GSTs in cell signaling without performing direct-binding experiments: Ishisaki et al. [64] have shown that increasing expression in GSTP1 protects against dopamine-induced apoptosis in dopaminergic neurons by decreasing JNK activity; Elsby et al. [56] have demonstrated an increase in the constitutive JNK signaling in mice lacking GSTpi; and overexpression of hGSTA2-2 protects against apoptosis in K562 cells [65]. More recently, Piaggi et al. [66] suggested that overexpression of GSTO1-1 is associated with activation of survival pathway (Akt, ERK1/2) and inhibition of apoptotic signaling (JNK) as well as protection against cisplatin-induced apoptosis. Among these studies, experiments done by Yin et al. [67] are particularly striking. Using a GSTPi inducible expression vector in 3T3 cells, they have shown that GSTpi allows protection against H_2_O_2_-induced cell death by coordinating an ERK/p38/IKK activation and a JNK suppression. 

## 5. S-Glutathionylation

S-glutathionylation is a posttranslational modification of proteins characterized by the conjugation of GSH to a low pKa cysteine residues allowing a protection against oxidative stress. Even if *in vitro* studies have underlined that this process occurs spontaneously, several studies have shown that GSTPi could influence the rate of this reaction [68–70]. Thus, Townsend et al. [68] have observed that, under stress conditions, GSTPi can mediate a self S-glutathionylation on its Cys-47 and Cys-101 and that these modifications by interfering with the GSTPi/JNK complex lead to GSTPi aggregate's formation and JNK activation. Two other papers have reported that GSTpi is able to S-glutathionylate 1-Cys-peroxiredoxin (1-Cys-Prx) [69, 70]. 1-Cys-Prx belongs to the nonselenoperoxidase family and catalyzed the degradation of hydroperoxides to alcohols. The 1-Cys-Prx has a thioredoxin fold in the N-terminal region where a conserved cysteine residue is involved in the peroxidase activity. The oxidized 1-Cys-Prx intermediate must react with another thiol compound to regenerate the sulfydryl cysteine of the active 1-Cys-Prx. In their experiments, Ralat et al. [69] have shown that GSTPi is able to interact with the oxidized form of 1-Cys-Prx and to re-activate this enzyme by glutathionylation. This glutathionylation is followed by the formation of an intermolecular disulfide bond between the two subunits. Then, the GSH-dependent reduction of the disulfide regenerates the reduced active-site thiol. 

Interestingly, the number of potential S-glutathionylated protein compared to the proteome is quite low as reported by Fratelli et al. [7] in hepatocyte after induction of an oxidative stress. However, more studies are necessary in order to understand the impact of GSTs on this post-translational modification and their role in the regulation of signaling pathway during oxidative stress. 

## 6. GSTs as a NO Carrier

NO is a short-life messenger playing a role in both physiologic (by activating the soluble guanylate cyclase) and cytotoxic processes (e.g., such as inflammation). Interestingly, many of these effects are linked to its ability to interact with Fe(II). In tumor cells, this mechanism resulted in a rapid diminution of energy and DNA synthesis due to the loss of iron-containing enzymes. Furthermore, several studies have shown that interactions with iron-sulfur cluster in proteins lead to their degradations and to the formation of dinitrosyl dithiol iron-complexes (DNICs). At physiological concentration, these complexes are suspected to play the role of NO carrier, increasing its half life, and suggesting that the concept of NO as a free diffusible compound in cells need to be reevaluated. Furthermore, recent studies have shown that MRP1 transporter is able to release these complexes from the cells [71]. On the other hand, at cytotoxic concentrations, such as during chronic inflammation, these complexes, by sequestrating NO, could prevent its cytotoxic effect. However, when the concentration becomes too important, the system is overhelmed and a toxicity occurs. For example, NO is able to bind iron and 2 glutathiones in order to form the dinitrosyl-diglutathionyl-iron complex (DNDGIC) [8]. This leads to a depletion in glutathion and could represent a key signal trigerring apoptosis. 

Several studies have shown that GSTs could bind DNICs. Thus GSTA1-1, GSTM1-1 and GSP1-1 are able to bind DNDGIC *in vitro* [72]. A crystal structure of the GSTP1-1-DNDGIC has even been obtained [73]. Tyr-7, in the active site of the GSTP1-1, coordinated to iron in DNDGIC displacing one of the GSH. More recently, Lok et al. [74] have suggested that GSTP1-1 acts to prevent NO-mediated iron released from MRP1 by sequestring DNICs. Thus a combinating effect of GSTP1-1 (storage of DNDGIC) and MRP1 (efflux of DNDGIC) seems to play a key role in cell protection against cytotoxicty.

## 7. Conclusion

Altogether, these observations clearly demonstrate that GSTs have roles beyond the simple detoxification reactions and seat themselves as crucial regulators of the stress kinase pathways. Among them, the GSTPi may be the most peculiar GST with its inhibitory role in various signaling pathways implicated in apoptosis or proliferation. Interestingly, GSTP1 is overexpressed in lung, ovary, pancreas, stomach, and colon cancers [1] and this high expression level has been correlated with resistance to several anticancer drugs. [75, 76]. In the light of the more recent works, interactions of GSTs with stress kinases could also be involved in such resistance mechanisms. Recently, Peklak-Scott et al. [77] concluded that the role of GSTP1-1 in cellular detoxification of cisplatin failed to totally explain resistance to this drug and that such mechanism should also involved the modulation of signaling pathways. Thus, strategies to prevent the apparition of multidrug resistance should aim at designing specific inhibitors able to disrupt interactions between GSTs and protein kinases. This approach has already been done by several authors [78–80]. However, in order to obtain these inhibitors, new studies are necessary to define the exact regions implicated in each interaction. On the other hand, GSTs role in the metabolism of endogenous compound such as 4-HNE or 15d-PGJ_2_ or in the S-glutathionylation of proteins also indicates that GST levels might be critical in the control of cell signaling. 

These specific functions of GSTs could lead to the development of new therapeutic approaches and to the identification of some interesting candidates for preclinical and clinical development. 

## Abbreviations

4-HNE: 4-Hydroxynonenal
15d-PGJ: 15-Deoxy-Δ-prostaglandin J
ARE: Antioxidant responsive element
ASK1: Apoptosis signal-regulated kinase 1
CDK: Cyclin dependent kinase
DNIC: Dinitrosyl iron-complex
DNDGIC: Dinitrosyl-diglutathionyl-iron complex
GSH: Glutathione
GST: Glutathione transferases
HBV-associated HCC: Hepatitis B Virus-associated Hepatocellular Carcinoma
IL-6: Interleukin 6
JNK: c-jun N-terminal kinase
MAPK: Mitogen-activated protein kinase
MRP: Multidrug Resistance Protein
NF-*κ*B: Nuclear factor *κ*B
NO: Nitric oxide
Nrf2: Nuclear factor-erythroid 2 p45-related factor 2
PPAR*γ*: Peroxisome proliferator-activated receptor *γ*
PG: Prostaglandin
ROS: Reactive oxygen species
TNF*α*: Tumor necrosis factor *α*
TRAF2: Tumor necrosis factor Receptor Associated Factor 2
Trx: Thioredoxin.
